# Intrinsic Capacity and Active and Healthy Aging Domains Supported by Personalized Digital Coaching: Survey Study Among Geriatricians in Europe and Japan on eHealth Opportunities for Older Adults

**DOI:** 10.2196/41035

**Published:** 2023-10-12

**Authors:** Vera Stara, Luca Soraci, Eiko Takano, Izumi Kondo, Johanna Möller, Elvira Maranesi, Riccardo Luzi, Giovanni Renato Riccardi, Ryan Browne, Sébastien Dacunha, Cecilia Palmier, Rainer Wieching, Toshimi Ogawa, Roberta Bevilacqua

**Affiliations:** 1 Medical Direction IRCCS, INRCA Ancona Italy; 2 Unit of Geriatric Medicine IRCCS, INRCA Cosenza Italy; 3 National Center for Geriatrics and Gerontology Obu City Japan; 4 Diocesan Caritas Association for the Archdiocese of Cologne Cologne Germany; 5 Clinical Unit of Physical Rehabilitation IRCCS, INRCA Ancona Italy; 6 Smart-Aging Research Center Tohoku University Sendai Japan; 7 Maladie d’Alzheimer Université de Paris Paris France; 8 Service de gériatrie 1&2, AP-HP, Hôpital Broca Paris France; 9 Institute for New Media & Information Systems University of Siegen Siegen Germany

**Keywords:** intrinsic capacity, functional ability, active and healthy aging, digital coaching, eHealth interventions, older adults

## Abstract

**Background:**

The worldwide aging trend requires conceptually new prevention, care, and innovative living solutions to support human-based care using smart technology, and this concerns the whole world. Enabling access to active and healthy aging through personalized digital coaching services like physical activity coaching, cognitive training, emotional well-being, and social connection for older adults in real life could offer valuable advantages to both individuals and societies. A starting point might be the analysis of the perspectives of different professionals (eg, geriatricians) on such technologies. The perspectives of experts in the sector may allow the individualization of areas of improvement of clinical interventions, supporting the positive perspective pointed out by the intrinsic capacity framework.

**Objective:**

The overall aim of this study was to explore the cross-national perspectives and experiences of different professionals in the field of intrinsic capacity, and how it can be supported by eHealth interventions. To our knowledge, this is the first study to explore geriatric care providers’ perspectives about technology-based interventions to support intrinsic capacity.

**Methods:**

A survey involving 20 geriatricians or clinical experts in the fields of intrinsic capacity and active and healthy aging was conducted in Italy, France, Germany, and Japan between August and September 2021.

**Results:**

The qualitative findings pointed out relevant domains for eHealth interventions and provided examples for successful practices that support subjective well-being under the intrinsic capacity framework (the benefits offered by personalized interventions, especially by promoting health literacy but avoiding intrusiveness). Moreover, eHealth interventions could be used as a bridge that facilitates and enables social engagement; an instrument that facilitates communication between doctors and patients; and a tool to enrich the monitoring actions of medical staff.

**Conclusions:**

There is an unexplored and significant role for such geriatric perspectives to help the development process and evaluate the evidence-based results on the effectiveness of technologies for older people. This is possible only when clinicians collaborate with data scientists, engineers, and developers in order to match the complex daily needs of older adults.

## Introduction

### Background

The challenge of supporting and promoting active and healthy aging (AHA) as “the process of developing and maintaining the functional ability that enables well-being in older age” [1] concerns the whole world. Within this AHA framework, “health” refers to physical, mental, and social well-being and “active” refers to continuing participation in social, economic, cultural, spiritual, and civic affairs. Functional ability is strictly connected to intrinsic capacity, the environment, and their interaction, and intrinsic capacity refers to the sum of an individual’s physical and mental abilities. Scientific data suggested that it is more valuable to focus on intrinsic capacity rather than on specific chronic diseases in older adults [2-13]. The worldwide aging trend requires conceptually new prevention, care, and innovative living solutions to support human-based care using smart technology. Indeed, technological progress is providing numerous hardware and software solutions to enable affordable AHA support and eHealth interventions for older adults in recent years [14-19]. Especially, digital coaching interventions seem to be promising [20-22]. For example, dialog systems and conversational user interfaces (also called conversational artificial intelligence [AI], voice user interface, and chatbot) allow natural interactions using dialog modeling techniques, and they are becoming ubiquitous nowadays [23-25]. Enabling access to AHA through personalized digital coaching services like physical activity coaching, cognitive training, emotional well-being, and social connection for older adults in real life could offer valuable advantages to both individuals and societies. The main challenge of such technologies is to secure independent living and prevention, helping older adults to live longer in their own homes with the possibility to act independently and participate actively in society. These functionalities are intended to support the continued well-being of users in the context of their health conditions, real-life situations, and needs. Moreover, smart systems provide particularly useful support for users with long-term therapy management challenges as it is required to maintain integrated well-being information management. These enable predictive analytics and thus alert services to minimize incidences of exacerbations of chronic conditions and allow their mitigation management so as to reduce the scale of emergency hospital admissions. At the same time, they contribute to providing guidance in everyday life for older users and contribute to better management of their physical, cognitive, social, or emotional frailty symptoms. Based on this, the complex phases of design, development, evaluation, and implementation of novel technologies, combining capabilities for integration into daily life routines and fulfilling personal and emotional needs, are all significant to elevate intelligent technologies to a more appropriate and sustainable AHA support for improving the overall well-being of older adults. This means that collaboration among different stakeholders, including designers, users, developers, etc, could form a “network of excellence” [26], enabling long-term involvement and co-creation of new concepts. Therefore, innovative approaches can support older adults in managing their health and well-being only if active collaborations are established between older adults, their informal and professional health carers, community members, and policy makers. Although a large amount of literature is available on older adults’ and caregivers’ perspectives of innovative technology, few studies have reported on clinicians’ views or their direct engagement in the design and development of such technologies [27,28].

Evidence on interventions designed to support the intrinsic capacity framework is of paramount relevance for older people, as they may allow personalized strategies to be put in place to support the independence and resilience of older people. However, numerous studies in the field have identified research trends and gaps that need to be solved to effectively integrate the intrinsic capacity framework with more traditional therapies and treatments [29].

The perspectives of health professionals and stakeholders are essential to understand not only how to design intrinsic capacity–integrated and technology-integrated interventions, but also how to assess the improvement in intrinsic capacity domains after conducting those interventions, as an assessment tool to identify improvements in intrinsic capacity as a whole (not only as a sum of domains) is still missing [29,30].

A starting point in this sense might be the analysis of perspectives from different professionals, like geriatricians, on such technologies. The perspectives of experts in the sector may allow the individualization of areas of improvement of clinical interventions, supporting the positive perspective pointed out by the intrinsic capacity framework, which is more in favor of a person-oriented approach than a disease-oriented approach, allowing the greater personalization of any clinical intervention. Therefore, the relevance of this study involves the identification of existing research trends and possible gaps with experts in the field, which should be applied in the near future when designing technology-based interventions.

### Objectives

The overall aim of this study was to explore the cross-national perspectives and experiences of different professionals in the field of intrinsic capacity, and how it can be supported by eHealth interventions (also named technology-based interventions or digital coaching). The specific research goals falling under this aim are as follows: (1) to collect information about which everyday practices can increase and sustain intrinsic capacity in the older population; (2) to identify what kind of technology could support the related practices; (3) to understand which technologies were useful to support the functional ability and quality of life of older people during the COVID-19 crisis; (4) to analyze the gender differences in planning interventions to support the intrinsic capacity and functional ability of older people, even considering eHealth interventions; and (5) to map the positive and negative aspects of the intrinsic capacity framework, even considering the possible roles of technologies. To our knowledge, this is the first study to explore the perspectives of geriatric care providers regarding technology-based interventions to support intrinsic capacity. Therefore, it contributes to the literature by demonstrating that there is an unexplored and significant role for such geriatric perspectives to help the development process and evaluate the evidence-based results on the effectiveness of technologies for older people. However, only when clinicians join forces with data scientists, engineers, and developers and when innovative technologies match the complex daily needs of older adults will we be able to exploit the full potential of these technologies [27]. This study is based on data collected during the e-VITA project that is aimed to improve well-being in older adults in Europe and Japan, thereby promoting active and healthy aging, contributing to independent living, and reducing the risks of social exclusion of older adults. In the eHealth intervention project, a European and Japanese Consortium collaborated to gain maximum outcomes and impacts for both regions, and to jointly develop and connect innovative smart living solutions that address the individual as well as cultural aspects and factors of the AHA framework and overall well-being through AI, intelligent data analysis, and tailored interventions based on information and communications technology (ICT) and real-life coaching.

### Cross-Sectional Approach in the e-VITA Project

Owing to demographic changes, decreasing numbers of care professionals, and dissolving family structures, all arguments are now turning toward digital solutions to support elderly care and prevention. Innovative approaches can support older people in managing their health and well-being; however, this is only possible if all beneficiaries are consulted and if they have sufficient ICT literacy and can access affordable and interoperable solutions that facilitate collaboration among older adults, their informal and professional health carers, community members, and policy makers.

Thus, it was planned to adopt a user-centered and value-sensitive participatory design approach in all phases of the e-VITA project, in collaboration with different stakeholders in all participating countries, considering their engagement as a crucial means to ensure the sustainability, acceptance, and uptake of e-VITA in European countries and Japan.

The exchange of good practices and the inclusion of stakeholders in all the countries will support local governments to adopt and adapt policies for AHA in Europe and Japan on a cross-national basis to build a reasonable support system that is sustainable as it fulfills the real needs of older adults, considering different cultural perspectives.

In the first phase, the cross-national approach has been applied to the analysis of the attitude toward virtual agents, such as e-VITA, in Italy, France, Germany, and Japan. Italy has a strong emphasis on family-based care for elderly people. Traditionally, Italian families take on the responsibility of caring for their aging relatives. However, with changing societal dynamics and increased mobility, the demand for formal care services has grown. The Italian government provides various support measures, such as financial aid and home care services, to assist families in caring for their elderly members. There are also nursing homes and assisted living facilities available for those who require more specialized care. In France, there is a comprehensive social welfare system that provides assistance to elderly people. The government operates a national health care system that includes coverage for long-term care services. Elderly individuals can receive home care services, such as help with daily activities, nursing care, and domestic assistance. Additionally, there are nursing homes and residential care facilities available for those who need more intensive care. The French government also provides financial aid and benefits to low-income seniors to ensure their well-being. Germany has a well-developed system of social protection for elderly people. The country has a mandatory long-term care insurance program that provides coverage for long-term care services. Elderly individuals can receive assistance with personal care, nursing, and household tasks through home care services. The German government also supports the development of community-based services and encourages independent living for seniors. Nursing homes and assisted living facilities are available for those who require round-the-clock care. In Japan, the care and support provided to elderly people are characterized by a combination of family involvement, community-based care, and formal care services. Similar to Italy, Japan places a strong emphasis on family-based care for elderly people. It is culturally expected that children will take care of their aging parents. Many elderly individuals live with their adult children or nearby, and families often provide primary support and assistance. Moreover, Japan has a well-developed system of community-based care services. Local governments and community organizations provide a range of services to support independent living for seniors. These services include home help, meal delivery, transportation assistance, social activities, and health monitoring.

## Methods

### Procedure

An essential phase of the e-VITA project is linked to the mapping of indicators and practices that facilitate stable good health conditions through consultation with relevant international experts in intrinsic capacity and AHA. Therefore, a survey involving 20 geriatricians or clinical experts in the fields of intrinsic capacity and AHA was conducted in Italy, France, Germany, and Japan between August and September 2021. The survey aimed to collect information on which everyday practices increase the intrinsic capacity and subjective well-being of older adults and how digital coaching can offer support in this process.

It was conducted using a semistandardized questionnaire formulated in English and then adapted to the language of the respective country (Italy, France, Germany, and Japan). The survey consisted of the following 5 topic sections populated by open questions (see Multimedia Appendix 1):

1. Topic 1. Clinical expert perspective: This section aimed to collect information about which everyday practices can increase and sustain intrinsic capacity in the older population (ie, everyday activities that older people can perform by themselves such as monitoring health vital signs, taking appropriate medication, lifestyle activities, etc).
2. Topic 2. Technology enhancement: In this section, experts can indicate what kind of technology they suggest for supporting the intrinsic capacity practice.
3. Topic 3. Supporting intrinsic capacity during COVID-19: This section focused on the identification of which technologies were found useful to support the functional ability and quality of life of older people by experts during the COVID-19 crisis.
4. Topic 4. Gender considerations: This section was devoted to analyze the gender differences in planning interventions to support the intrinsic capacity and functional ability of older people.
5. Topic 5. Aspects of intrinsic capacity: This closing open question aimed to gather information on the positive and negative aspects of the intrinsic capacity framework, and the improvements and future areas of interventions to support older people.

An invitation email was sent to invite the experts and to explain the objectives of the project as well as the aim of the survey. An informed letter was attached to the email, and the experts were kindly invited to visit the project website [31] as a repository of all the significant content, videos, podcasts, press releases, and materials related to the project activities.

### Ethical Considerations

The procedures and approaches adopted during all design activities of the e-VITA project are in accordance with the ethical standards and have been approved by the Ethics Committee of Siegen University (ER_31/2021; June 21, 2021).

Sensitive and private data were covered by informed consent in each participant’s own language, in accordance with the ethical standards on human experimentation (institutional and national), the GDPR (General Data Protection Regulation) 2018, and the national legislations on privacy and data protection.

### Recruitment

The experts recruited for the survey in Germany are network partners of the Diocesan Caritas Association for the Archdiocese of Cologne. They were contacted by email. The email explained the aims of the survey and contained the questionnaire and an informed consent form. The informed consent form was filled out digitally by the study participants and returned together with the questionnaire.

In France, the experts were recruited through the Broca Living Lab network. An email invitation was sent to the experts. The email explained the objectives of the project and the purpose of the questionnaire, and contained the questionnaire. If they agreed to participate, the experts returned the completed questionnaire by email.

In Italy, the experts were recruited within the hospital units of the National Institute of Health and Science on Aging* *(INRCA). An invitation email was sent to invite the experts. The email explained the objectives of the project and the aims of the survey. If they agreed to participate, they signed the informed consent form attached to the email and answered the questions on the questionnaire. All participants returned the signed informed consent form and the questionnaire by email.

In Japan, the experts were recruited within the National Center for Geriatrics and Gerontology (NCGG) and through the network of the eHealth intervention Japanese partner. An invitation email was sent to invite the experts. The email explained the objectives of the project and the aims of the survey. If they agreed to participate, they signed the informed consent form attached to the email and answered the questions on the questionnaire. All participants returned the signed informed consent form and the questionnaire by email. In total, 5 experts were recruited.

Individuals who took part in the study were provided with and asked to sign a written informed consent form regarding data.

### Statistical Analysis

Data were collected and analyzed in the native language of each site, and then, the local results were translated into English and combined cross-nationally. Local questionnaires and the national findings were analyzed using the framework analysis method [32,33]. The MAXQDA software package (VERBI Software GmbH) for qualitative research was used. Researchers classified and categorized text data segments into a set of codes that were then combined under main themes. Specifically, different data segments were associated with the same code, and codes were gathered under the same theme. To ensure comparability among the countries and their languages, the main categories were created deductively based on the topics of the questionnaire used (Topics 1-5) for gathering insights in the fields of intrinsic capacity and AHA. The substantive differentiation of these topics was carried out inductively in the further course of the analysis on the basis of the raw data from the participants for the open questions. To validate the data analysis, the lead author (VS) performed the coding. Then, the list of codes was validated by 2 other researchers from the team (JM and ET) in order to minimize personal bias and interpretation errors, and to ensure validity and reliability. Quotes were then sorted out, and comparisons were made between them [34]. Following this coding process, quotations referring to the same quotes were grouped in a code, and the results have been reported using the frequency of codes (see Multimedia Appendix 2) as the number of times each code was found in the case of general agreement (ie, n/20). Specific quotations have been reported to clarify the meaning of the codes merged from the analysis.

## Results

### Study Population

A total of 20 experts in the field were recruited (4 from France, 5 from Italy, 5 from Japan, and 6 from Germany). However, 1 questionnaire had to be excluded from the qualitative analysis of the open-ended questions owing to missing answers. Table 1 shows the main characteristics of the respondents.

The different topics are presented below. The most frequent sets of codes and findings are discussed in each paragraph and reported in Multimedia Appendix 2.

**Table 1:** Participant characteristics.

Participant ID^a^		Gender	Specialization
DE_EXP_01		Male	Elderly care, nursing science
DE_EXP_02		Male	Nursing administrator/nursing manager, Master of Gerontomanagement
DE_EXP_03		Female	Specialist adviser for elderly care
DE_EXP_04		Female	PhD
DE_EXP_05		Female	Health Care Research MSc, Doctorate in Health Science (degree pending)
DE_EXP_06		Female	Outpatient care and palliative care
FR_GER_01		Female	Geriatrician
FR_GER_02		Female	Geriatrician
FR_EXP_03		Female	Psychologist
FR_EXP_04		Male	Psychologist
IT_GER_01	Male		Geriatrician
IT_GER_02	Male		Geriatrician
IT_GER_03	Female		Geriatrician
IT_GER_04	Male		Geriatrician
IT_GER_05	Male		Geriatrician
JP_GER_01	Male		Geriatrician
JP_GER_02	Male		Geriatrician
JP_GER_03	Male		Geriatrician
JP_EXP_04	Male		Rehabilitation medicine
JP_GER_05	Male		Geriatrician

^a^Regarding the participant ID, DE refers to Germany, FR refers to France, IT refers to Italy, JP refers to Japan, EXP refers to clinical expert, and GER refers to geriatrician.

### Topic 1: Clinical Expert Perspective

Across all countries, the experts agreed on the value of physical (18/20, 90%) and social activities (14/20, 70%) as the main everyday practices that can increase and sustain the intrinsic capacity of older people. Indeed, all physical activities like any kind of sports (walking, jogging, swimming, cycling, yoga, etc) as well as the independent performance of household chores and the active pursuit of hobbies can prevent frailty symptoms and preserve physical functioning. Some responses were as follows:

In ageing research, the relevance of physical activity is very often pointed out. The empirical studies to date show that regular exercise, for example, reduces the risk of developing frailty symptoms (e.g. weight loss, fatigue), which in my view greatly influences a person's intrinsic capacity.

Participant ID: DE_EXP_04

The promotion of lifestyle activities is mandatory to avoid physical decompensation and to sustain physical and mental health.

Participant ID: IT_GER_04

Moreover, nutrition as well as a healthy and balanced diet (7/20, 35%) was a significant determinant to have enough energy and strength to participate in physical activities. One participant commented as follows:

All the physical activities empower social activities such as going to the cinema, the theatre, the interactions among individuals, family members, and health care professionals.

Participant ID: IT_GER_05

…having dinner with friends or family, looking after grandchildren, being an active member of an association.

Participant ID: IT_GER_05

Other ranked activities cited by half of the European experts were cognitive activities (9/20, 45%) related to intellectual and affective-relational stimuli and self-care (5/20, 25%). European and Japanese geriatricians (9/20, 45%) underlined how regular health status monitoring is important to identify any deficits or medical problems at an early stage and thus perform appropriate interventions. In this specific case, health literacy was seen as basic knowledge for self-management.

### Topic 2: Technology Enhancement

According to the respondents, physical (13/20, 65%), cognitive (10/20, 50%), and social (9/20, 45%) interventions based on technology could improve physical activities and social connections, as well as self-esteem and well-being. This improvement could lead to a general overcoming of the anxiety in performing daily activities that may contribute to the sensation of mental fatigue. For example, the respondents imagined that technology-based interventions could track adequate nutrition and hydration using a daily diary (9/20, 45%) and physical activities using geolocalization tools (5/20, 25%) and reminders.

These tracking measures could also be shared with other older adults to exchange experiences and support each other as in a virtual community. This could lead to approaching the need for social connections at the same time. Indeed, integrating eHealth interventions into everyday life or using programs to create interactions between humans and virtual coaches could help people fulfill their wishes and enjoy their hobbies. Based on their clinical experience, the experts underlined that individuals often do not know exactly what they like, so it is essential to implement a function that proposes a list of enjoyable activities and assists them in performing the activities. Indeed, the importance of offering support, motivational tips, and suggestions was underlined (5/20, 25%) as a key to fostering positive engagement. Moreover, the service should be personalized (5/20, 25%) considering that due to the heterogeneity of the target, some older adults prefer to be “active” users who maintain control and want the feeling of power, while others require more direction.

Moreover, the use of virtual coaches, smartphones, and computers would facilitate these activities. Indeed, these devices would make it possible to centralize different applications covering broad areas, for example, by offering gymnastic applications to strengthen muscular capacities and encouraging outdoor activities (5/20, 25%), which can then improve social links. These tools also help to promote cognitive abilities by proposing personalized activities that can be close to home or remote, such as visiting virtual museums. Additionally, user autonomy can be encouraged through calendars, lists, and banking applications.

Regarding e-coach interventions, some experts (4/20, 20%) underlined the importance of having the easiest interface as a significant component of any kind of technology designed for older adults. This includes the use of clearly understandable icons and control through voice interaction as the most natural way of communication. In addition, suggestions were made to combine technical and human support.

For example, 1 participant proposed that supermarkets should be geared to the needs of older people and that shopping should be planned using apps:

Supermarkets for older people with products at eye level and large numbers, checkouts with friendly staff who take their time. Apps that suggest products to order through pictures.

Participant ID: DE_EXP_05

Moreover, physical training should be promoted by supporting technologies where appropriate. One participant commented as follows:

…exercises to strengthen the muscles, possibly guided by artificial intelligence (AI) and measurement of the muscle tissue to monitor success.

Participant ID: DE_EXP_06

To maintain mobility, the experts also suggested using technology to make trips more predictable. This includes the identification of possible barriers that the user might encounter on the way, the use of public transport, and the knowledge of public toilets.

To promote social participation in the community, the experts advised social networks to connect users (possibly with similar interests) and mention where people can offer their help in specific areas (9/20, 45%). One participant stressed the concept of technologies that cooperate with clinicians:

I cannot imagine a future in which any electronic device or e-coach will replace humans in supporting older patients. I think that an extremely useful action would be to implement technology‐based services delivered and managed by healthcare and other professionals. To note, I am not saying that technology is not important but some “human functions” cannot be replaced.

Participant ID: IT_GER_03

### Topic 3: Supporting Intrinsic Capacity During COVID-19

During the COVID-19 emergency, the surveyed participants made extensive use of video communication and social apps to establish contact and opportunities with their patients (20/20, 100%). In Germany, such platforms were used even for online education to support the functioning and quality of life of older people during the lockdown. Indeed, these tools allowed older adults to maintain social links and even promoted physical activities. All the experts reported that the use of such remote modalities was very important to decrease the sense of social isolation, uncertainty, and powerlessness during the period of social distancing. This represented a very important tool to increase mental well-being and social engagement.

### Topic 4: Gender Considerations

The surveyed participants underlined that a virtual coach should account first for individual differences and then, in some cases, for gender (12/20, 60%). The experts usually planned their interventions by accounting for individual differences (characteristics and preferences of the person). Nine of them suggested that empathetic communication, discussions or reactions involving the user, and the different physical abilities between men and women should be definitely accounted for, considering gender differences. Moreover, the user could choose the gender of the coach. The participants commented as follows:

I'm not sure if there are really gender differences or if they don't break down more and more in the next years and one should pay much more attention to the individuality of the person and generalized approaches based on gender characteristics don't lead to achieving the outcome. Technological support for crochet can also be interesting for men.

Participant ID: DE_EXP_01

…make sure to personalize the intervention according to the patient's wishes and interests, their previous lifestyle.

Participant ID: FR_GER_01

Make a fair assessment. No gender difference but depends on the person's abilities/personalities/ understanding interests.

Participant ID: FR_EXP_03

One participant described that gender-specific differences should be considered especially against the background of age, culture, and religion:

A distinction must be made here depending on age and also in relation to culture. A gender-specific consideration is necessary around age, as gender-related attributions still play a major role here. e.g. for women, domestic activities and handicrafts, and for men, e.g. playing Skat, handicraft-related tasks, early morning hopping. The younger the people are, the more the activities are similar. In general, however, there is always a cultural and religious distinction.

Participant ID: DE_EXP_06

Another participant commented as follows:

Even though women were considered at risk of increased functional capacity with aging, today we are seeing how such differences are becoming less evident, thanks to improvement of lifestyles and social engagement of women.

Participant ID: IT_GER_04

The following specific suggestions regarding the virtual coach were shared: the language and choice of words could be individually adapted by the user, the gender of the coach could be individually selected, and the difference in physical abilities between men and women should be taken into account.

The importance of communicating the information differently was mentioned:

Women are often more receptive to prevention advice. For men it seems more important that they understand the direct individual benefit of interventions. When men are in a couple, it is sometimes interesting to go through his wife to get the messages across.

Participant ID: FR_GER_02

Participant FR_EXP_04 explained that people are addressed and interventions are planned according to the “bio-psycho-social level. From a cultural and developmental point of view,” which explains why sometimes the interventions are not the same according to gender.

Other geriatricians commented as follows:

Preservation of intrinsic capacity needs to be particularly targeted in women, since they are more prone to develop functional disability over time compared to men of the same age.

Participant ID: IT_GER_05

Women are more prone to locomotion and cognitive decline, and prefer group activities, while men have general characteristics such as a preference for solo activities, and it is necessary to understand these characteristics while giving consideration to individuality.

Participant ID: JP_GER_01

Regarding physical activities probably men and women should practice more different domains (eg, balance vs strength). Women are more prone to socialization and less close to accept a device like that (I suppose). When trying to implement empathetic discussions or reactions to the user, gender should be definitely accounted for.

Participant ID: IT_GER_03

### Topic 5: Aspects of Intrinsic Capacity

The participants commented as follows:

The intrinsic capacity framework is full of positive aspects. Indeed, it may improve self-wellbeing through enhancement and preservation of baseline capacities. It would be important to provide patients with appropriate tools and technologies to support choices and to speed up the recognition of individuals at risk of decline.

Participant ID: IT_GER_04

The intrinsic capacity Framework pushed the importance of prevention compared to treatment and of the preservation of physiological reserve compared to the disease-based approach.

Participant ID: IT_GER_05

The advantages of the concept were recognized as follows:

It considers the person as a whole, ie, psychological as well as physical aspects are included.

Participant ID: DE_EXP_04

However, the same expert also stated the following:

The concept of intrinsic capacity is based on the assumption that all persons have the same (financial, social) resources to maintain or improve their own intrinsic capacity and mentions that it is often described in the literature as having been developed for the “privileged group” of older persons.

Participant ID: DE_EXP_04

This is particularly significant in light of the fact that research shows that social and environmental contexts lead to negative health outcomes. Therefore, the development of the e-coach should also take into account people who, for example, have limited financial resources or few digital or health-related skills.

For participant FR_GER_02, intrinsic capacities are important to take into account because their decline leads to a loss of autonomy. However, for this expert, these intrinsic capacities are sometimes complicated to understand for elderly people:

Intrinsic capacity's definition and evaluation are complicated for the elderly.

Participant ID: FR_GER_02

Another participant commented as follows:

However, this concept is often difficult to understand by the elderly because the decline in intrinsic capacities sometimes goes unnoticed and is only little taken into account by carers. It is also little sought after by carers who are often focused on the loss of autonomy: It would be appropriate to better communicate and raise awareness of the concept of intrinsic capacities among older people and carers.

Participant ID: JP_GER_01

The promotion could be valuable since “the positive aspects of intrinsic capacity are related to health and longevity and are useful for self-management” [Participant ID: JP_GER_01] and “many elderly people recognize that maintaining their intrinsic abilities is in itself a positive aspect” [Participant ID: JP_GER_03].

Another participant commented as follows:

In terms of improvements and future areas of intervention to support older people, the implementation of the intrinsic capacity framework should ensure to sensitize both health care professionals and individuals on the importance of intrinsic capacity and functional capacity.

Participant ID: IT_GER_05

This comment indicates that the promotion of intrinsic capacity is intended for not only older adults and their caregivers but also all clinical staff.

Moreover, 1 participant stated:

A fair assessment of the functional capacities and the impact on the person's life should refer to the functional abilities and the impact of their loss on the older person, as some people will accept and regulate their living conditions, while others will suffer and may increase this loss.

Participant ID: FR_EXP_03

If there is a negative aspect to intrinsic capacity, “it is spiritual distress over the fact that actual physical functions are declining compared to the ideal or desired physical functions” [Participant ID: JP_GER_03].

On the other hand, another participant commented as follows:

Intrinsic capacity is the capacity or ability of an individual person, and a person's sense of value. We had better think of it as a positive or negative aspect, but rather as a way to capture individual abilities.

Participant ID: JP_GER_05

Furthermore, how intrinsic capacity could be supported by a virtual coaching system is seen in the fact that it could improve the general well-being of users, potentially support people to live independently at home for as long as possible, and support their health safety. The participants commented as follows:

It could offer a multitude of activities concerning cognitive stimulation (watching reports/films, learning a foreign language), physical activity (learning to dance, relaxation) and promoting social contacts (web conferencing, teleconsultation). And, the virtual coaching system should take into account ethical considerations, in particular the fact of not infantilizing, of respecting the user's free will, the user's capacity to make choices and of proposing personalized interventions since a human is by definition not a machine, he does not behave in a uniform manner. This system is disturbing in its reduction of humanity to a norm.

Participant ID: FR_EXP_04

It is questionable whether every person can afford this technology. In addition, isolation in a technology-infused home could occur and social contacts could be pushed into the background.

Participant ID: DE_EXP_03

The interest of the users could decrease and that suggestions from the coach are not accepted or are perceived as annoying.

Participant ID: DE_EXP_05

## Discussion

### Principal Findings

This study aimed at identifying and analyzing (1) which everyday practices can increase and sustain intrinsic capacity in the older population; (2) what kind of technology could support the related practices; (3) which technologies were found useful to support the functional ability and quality of life of older people during the COVID-19 crisis; (4) the gender differences in planning interventions to support the intrinsic capacity and functional ability of older people, even considering eHealth interventions; and (5) which are the positive and negative aspects of the intrinsic capacity framework, even considering the possible roles of technologies.

The qualitative findings point out relevant domains for eHealth interventions and provide examples for successful practices that support subjective well-being under the intrinsic capacity framework. From Topic 1 (clinical expert perspective), participants agreed on the value of physical and social activities as the main everyday practices that can increase and sustain the intrinsic capacity of older people. The relevance of supporting physical activities and social connectedness, especially through technology, is in line with the literature in the field of multicomponent interventions to support older people’s well-being, as demonstrated by large clinical studies, such as MyAHA [7], FINGER (Finnish Geriatric Intervention Study to Prevent Cognitive Impairment and Disability) [12,13], and MAPT (Multidomain Alzheimer Preventive Trial) [10,11]. All those studies, in fact, have provided promising insights on the benefits offered by personalized interventions aimed at targeting multiple domains of intrinsic capacity, such as physical activity, cognitive stimulation, and participation in social life, in terms of improvement of the overall health status and specific health domains.

Moreover, in the results of Topic 1, it emerged that the eHealth intervention could promote health literacy. Since low health literacy is associated with worse health outcomes [35-37], the eHealth intervention could also promote knowledge and skills to improve the health literacy of older people. For example, the experts cited healthy lifestyle and nutrition knowledge or skills for managing possible diseases. From Topic 2 (technology enhancement), it emerged that the eHealth intervention must be personalized and matched with the users’ needs since the “one-size-fits-all” approach is not suited to a diverse set of users. The challenge is to adapt content and functionalities to the aims, behaviors, preferences, context, and lifestyle of the intended user. This means changing the system’s functionalities, interface, content, or distinctiveness to increase its personal relevance. It is indeed one of the most critical issues that emerged in other studies [38-40]. Another implication related to the concept of personalization is that the eHealth intervention must promote independence and personal control. Any services or functionalities should be controlled by the users who, at every point in time, can decide if they are able or unable to manage them. Older adults are not a homogeneous group, and interindividual variability (between individuals) and intraindividual variability (within individuals) need to be taken into account. For example, the eHealth intervention could support seniors in maintaining their abilities and could make them feel as active and determined as possible, increasing their control over the world [41-44]. For sustainable interventions, digital coaching should propose and encourage activities in accordance with one’s own values. Even if some experts underlined that often individuals do not know what they like or need to be pushed to do some activities, technology-based coaching should not impose but rather propose interventions for older adults and should always avoid intrusiveness by respecting the privacy and will of the person concerned. Another important vision that emerged from Topic 2 and was reinforced by Topic 3, supporting intrinsic capacity during COVID-19, was that the eHealth intervention could also be intended and used as a bridge that facilitates and enables social engagement and relationships. Digital coaching could have the ability to enhance and enrich the lives of older adults by facilitating (not substituting) better interpersonal relationships, thus making it easier to connect with loved ones, friends, and the community, as well as mitigating loneliness and isolation. For example, as proposed by the majority of experts, the eHealth intervention should encourage sharing experiences on emotional well-being and connectedness among older adults. Definitely, the level of social engagement and the sense of belonging as a social member of the community have significant impacts on active and healthy aging in place [45-50]. Despite this important role of enabling social connections, within Topic 5 (aspects of intrinsic capacity), it was mentioned that digital interventions should avoid the risk of isolation in a technology-infused home. This recommendation points out the importance of not substituting human companionship and real-life relationships with AI. On the contrary, technology-based interventions could provide a link with medical staff as reported in the findings of Topic 2. The system could be seen as an instrument that facilitates communication between doctors and patients, as well as a tool to enrich the monitoring of actions by medical staff, for example, in the case of regular health status checking to identify any deficits or medical problems at an early stage and perform appropriate interventions. The experts underlined the necessity to develop a system that guarantees a higher level of usability and learnability since older adults are not technology native and they could sometimes have difficulties in approaching new devices. For instance, in Topic 2, they suggested the enrollment of caregivers or relatives as facilitators of acceptance and engagement with the system. For people living alone, an additional “human” support may be beneficial. Moreover, guaranteeing equal access to all individuals must be a precondition.

From the main results of Topic 4 (gender considerations), the experts enrolled in this study explained how they usually plan their interventions by accounting for individual differences (characteristics and preferences of the person). They suggested that empathetic communication, discussions or reactions involving the user, and the different physical abilities between men and women should be definitely accounted for, considering gender differences.

Various practices were analyzed from a gender perspective through the literature. For example, the high frequency of social participation activities is an important driver of well-being and better health for older women and men, and should be a focus of any technology-based intervention. Even if older men are less involved in social activities, they are more sensitive to loneliness and tend to need a closer social circle [51]. Another issue to consider is that women have a higher life expectancy than men with related consequences. They are living alone longer with less incomes and more health needs. This is one of the drivers for women to live with family members. Physical activities and good nutrition are necessary to maintain a healthy life and high well-being among older people. When designing an eHealth coaching system for older adults who want to age in place following an active and healthy aging life, these specific needs of women and men need to be recommended.

The positive effect of physical activities on the health and well-being of older people, regardless of gender, is a known factor, and technologies should therefore be a support in the practice of the activities, offering different formulas and regular monitoring of the completion of the activities. For women, the e-coach system could look at the internal motivations (completion of a psychological need), and for men, it could focus on external motivations such as the competitive aspect and playing sports in a group [52,53]. Finally, the educational level is also a motivating factor for performing sports. eHealth interventions should take this into consideration to make future generations and public institutions aware of the importance of a long-term vision of sports education for the well-being of older people, especially older women [54]. Moreover, users could choose the gender and the characteristics of the coach as reported in previous studies mentioning the importance of design features, including the agent’s age, gender, and role [55,56].

Intrinsic capacity can be supported by digital interventions to improve the general well-being of users, but a great effort is needed to promote the concept among clinicians, caregivers, and patients. The goal of supporting the intrinsic capacity of individuals through eHealth interventions is an important one, as it enables people to maintain and enhance their physical and mental abilities, promoting healthy aging and overall well-being. eHealth interventions refer to the use of electronic means, such as digital platforms, mobile apps, and remote monitoring devices, to deliver health care services, information, and support. It is crucial to ensure that eHealth interventions are accessible, user friendly, and tailored to the specific needs and capabilities of the target population. Regular evaluation and feedback from users and health care professionals can help refine and improve these interventions over time. By leveraging the power of technology, eHealth interventions have the potential to effectively support and enhance the intrinsic capacity of individuals, empowering them to lead healthier and more independent lives [29]. Nowadays, in fact, there is a lot of evidence highlighting the positive role of eHealth sociotechnical interventions in providing support to intrinsic capacity domains, such as cognitive status, locomotion, energy consumption and nutrition management, attention to reduced vision and hearing capacity, and psychological support. Despite this, standardized training on eHealth literacy [57] and a comprehensive assessment tool to evaluate the impact on intrinsic capacity are still lacking [20,29]

### Limitations

While this study benefitted from including a survey for professionals across Europe and Japan, linguistic barriers may have limited the quality of the survey. The data collection was conducted in the national language, and then, the data were translated from the native language to English. Thus, the results could have been impacted by this language switch. The use of software programs for qualitative analyses counteracted this issue. Despite this, the survey captured the main reflections and experiences of the participants from cross-national and multi-language perspectives. On the contrary, the specific national centrality of Italy, France, Germany, and Japan, as well as gender disparities in sample size could have introduced biases and could be seen as significant limitations that do not allow for the generalization of the results. Furthermore, the use of qualitative analysis in combination with a semistandardized questionnaire instead of a mixed-methods approach, which could have guaranteed a broad understanding of thoughts and emotions toward the use of eHealth interventions, could be considered another limitation of this study. The open-ended questions in the questionnaire provided only limited scope for answers, and in-depth enquiries with the experts were not possible owing to the design. Moreover, the data collection was performed during the pandemic period across all countries, and this situation could have impacted the thoughts and emotions of the respondents. Another limitation is related to the sample size. Although there is no commonly accepted sample size in qualitative studies, in this research, purposive sampling [58-60] was used since the researchers were interested in informants having the best knowledge of the intrinsic capacity field, which is a research topic still in its infancy. Furthermore, the data analysis demonstrated saturation of the findings for the linked items and their categorization [61,62] after applying the investigator triangulation method [63] with 3 members of the research team studying and evaluating the data from the source. All these methodological steps meet the trustworthiness criteria [64,65].

### Conclusions

This research study explored the opportunities of eHealth interventions designed for older adults according to the perspectives of a sample of professionals in the fields of AHA and intrinsic capacity from Europe and Japan. Indeed, digital coaching seems to be very promising and able to sustain physical activity coaching, cognitive training, emotional well-being, social connection, etc, for older adults in real life. These multicomponent interventions could offer valuable advantages to both individuals and societies, and therefore help in mitigating the need for new prevention, care, and innovative living solutions, as already highlighted by the results collected with older people in a similar study [66].

Through the qualitative analysis discussed in this study, the importance of the perspective of experts was underlined to demonstrate that there is an unexplored and significant role for such a geriatric perspective to help the development process and to evaluate the evidence-based results on the effectiveness of technologies for older people. This is possible only when clinicians collaborate with data scientists, engineers, and developers in order to match the complex daily needs of older adults. This synergy needs to be driven, and there is an urgent need to strengthen data, research, and innovation to accelerate the implementation of eHealth interventions in order to meet the interests and needs of the aging society, especially with the hindsight of the COVID-19 pandemic, which revealed the need for improvement in all health services.

Future developments should include the involvement of all stakeholders in the service, such as older adults as potential users and technical or digital health experts in geriatric care. Moreover, implementation approaches and recommendations to specifically translate the conclusions of the study should be planned.

## Appendix

Multimedia Appendix 1 Semistandardized questionnaire.

Multimedia Appendix 2 Feedback from experts across countries.

## Abbreviations

AHA: active and healthy aging
AI: artificial intelligence
ICT: information and communications technology
