# Supplementary material for: Intrinsic Capacity and Active and Healthy Aging Domains Supported by Personalized Digital Coaching: Survey Study Among Geriatricians in Europe and Japan on eHealth Opportunities for Older Adults
Source: J Med Internet Res. 2023 Oct 12;25:e41035. doi: 10.2196/41035 (PMC10603559; doi:10.2196/41035)
Supplement: Multimedia Appendix 2 [file jmir_v25i1e41035_app2.docx]

**Multimedia Appendix 2.** Feedback from experts across countries.

| **Topic** | **Code** | **Germany**  **(N=6)** | **France**  **(N=4)** | **Italy**  **(N=5)** | **Japan**  **(N=5)** | **Frequency**  **(x/20)** |
| --- | --- | --- | --- | --- | --- | --- |
| Clinical expert perspective | Physical Activity | DE_EXP_01 DE_EXP_02  DE_EXP_03  DE_EXP_04 | FR_GER_01  FR_GER_02 FR_EXP_03  FR_EXP_04 | IT_GER_01 IT_GER_02  IT_GER_03 IT_GER_04  IT_GER_05 | JP_GER_01 JP_GER_02  JP_GER_03 JP_EXP_04 JP_GER_05 |  |
|  |  | 4/6 | 4/4 | 5/5 | 5/5 | 18/20 |
|  | Social Activity | DE_EXP_01 DE_EXP_03 DE_EXP_05 | FR_GER_01 FR_GER_02 FR_EXP_03 FR_EXP_04 | IT_GER_01 IT_GER_02 IT_GER_03  IT_GER_04 | JP_GER_01 JP_GER_02 JP_GER_03 |  |
|  |  | 3/6 | 4/4 | 4/5 | 3/5 | 14/20 |
|  | Cognitive Activity | DE_EXP_02  DE_EXP_02 | FR_GER_01  FR_GER_02  FR_EXP_03 | IT_GER_01  IT_GER_02  IT_GER_03  IT_GER_04 |  |  |
|  |  | 2/6 | 3/4 | 4/5 |  | 9/20 |
|  | Health monitoring | DE_EXP_02  DE_EXP_06 | FR_GER_02  FR_EXP_04 | IT_GER_02  IT_GER_03  IT_GER_05 | JP_GER_01  JP_GER_02 |  |
|  |  | 2/6 | 2/4 | 3/5 | 2/5 | 9/20 |
|  | Self Care | DE_EXP_03  DE_EXP_06 | FR_EXP_04 | IT_GER_01  IT_GER_02 |  |  |
|  |  | 2/6 | 1/4 | 2/5 |  | 5/20 |
| Technology enhancement | Physical Intervention based on technology | DE_EXP_01  DE_EXP_02  DE_EXP_03 | FR_GER_01  FR_EXP_03  FR_EXP_04 | IT_GER_01  IT_GER_02  IT_GER_03  IT_GER_04  IT_GER_05 | JP_GER_02  JP_GER_03  JP_EXP_04 |  |
|  |  | 3/6 | 2/4 | 5/5 | 3/5 | 13/20 |
|  | Cognitive intervention based on technology | DE_EXP_01  DE_EXP_03  DE_EXP_05 | FR_EXP_03  FR_EXP_04 | IT_GER_02  IT_GER_03  IT_GER_04  IT_GER_05 |  |  |
|  |  | 3/6 | 2/4 | 5/5 |  | 10/20 |
|  | Social intervention based on technology | DE_EXP_01  DE_EXP_02  DE_EXP_03  DE_EXP_05 |  | IT_GER_03  IT_GER_04  IT_GER_05 | JP_GER_01  JP_GER_02 |  |
|  |  | 4/6 |  | 3/5 | 2/5 | 9/20 |
|  | Nutrition, water intake diary | DE_EXP_02 | FR_GER_02  FR_EXP_03  FR_EXP_04 | IT_GER_02  IT_GER_05 | JP_GER_02  JP-03  JP-05 |  |
|  |  | 1/6 | 3/4 | 2/5 | 3/5 | 9/20 |
|  | Support, suggestions and motivation | DE_EXP_01 | FR_GER_02 | IT_GER_02  IT_GER_03 | JP_GER_02 |  |
|  |  | 1/6 | 1/4 | 2/5 | 1/5 | 5/20 |
|  | Personalization | DE_EXP_01 |  | IT_GER_02  IT_GER_03  IT_GER_05 | JP_GER_03 |  |
|  |  | 1/6 |  | 3/5 | 1/5 | 5/20 |
|  | Gymnastics applications | DE_EXP_01  DE_EXP_04 |  | IT_GER_01  IT_GER_04 | JP_GER_03 |  |
|  |  | 2/6 |  | 2/5 | 1/5 | 5/20 |
|  | Geolocalization |  | FR_GER_01  FR_GER_02 | IT_GER_01  IT_GER_02 |  |  |
|  |  |  | 2/4 | 2/5 |  | 4/20 |
|  | Easy interface | DE_EXP_02 |  | IT_GER_03  IT_GER_05 | JP_GER_02 |  |
|  |  | 1/6 |  | 2/5 | 2/5 | 4/20 |
| Supporting IC during COVID-19 | Video conference tools | DE_EXP_01  DE_EXP_02  DE_EXP_03  DE_EXP_04  DE_EXP_05  DE_EXP_05 | FR_GER_01  FR_GER_02  FR_EXP_03  FR-04 | IT_GER_01  IT_GER_02  IT_GER_03  IT_GER_04  IT_GER_05 | JP_GER_01  JP_GER_02  JP_GER_03  JP_EXP_04  JP-05 |  |
|  |  | 6/6 | 4/4 | 5/5 | 5/5 | 20/20 |
| Gender considerations | Other factor are more important (age, educational level, etc) | DE_EXP_01  DE_EXP_02  DE_EXP_04  DE_EXP_05 | FR_GER_01  FR_EXP_03  FR_EXP_04 | IT_GER_01  IT_GER_02 | JP_GER_02  JP_EXP_04  JP_GER_05 |  |
|  |  | 4/6 | 3/4 | 2/5 | 3/5 | 12/20 |
|  | Specific gender differences to take into account | DE_EXP_02  DE_EXP_03  DE_EXP_04 | FR_GER_02 | IT_GER_03  IT_GER_04  IT_GER_05 | JP_GER_01  JP_GER_03 |  |
|  |  | 3/6 | 1/4 | 3/5 | 2% | 9/20 |
