# Supplementary material for: Intrinsic Capacity and Active and Healthy Aging Domains Supported by Personalized Digital Coaching: Survey Study Among Geriatricians in Europe and Japan on eHealth Opportunities for Older Adults
Source: J Med Internet Res. 2023 Oct 12;25:e41035. doi: 10.2196/41035 (PMC10603559; doi:10.2196/41035)
Supplement: Multimedia Appendix 1 [file jmir_v25i1e41035_app1.docx]

**Multimedia Appendix 1.** Semistandardized questionnaire

Dear Prof/Dr.

Thank you for accepting to answer to the E-Vita Experts’ Survey! In this survey, the following definitions are used as reference points:

**“Intrinsic capacity”** comprises all the mental and physical capacities that a person can draw on and includes their ability to walk, think, see, hear and remember. The level of intrinsic capacity is influenced by several factors such as the presence of diseases, injuries and age-related changes.

**“Functional ability”** consists of the intrinsic capacity of the individual, relevant environmental characteristics and the interaction between them.

Based on the following framework, please answer the following questions. Your experience is of utmost importance for our research.

This questionnaire was devised to clarify how to improve the intrinsic capacity of older adults.

Date of filling out:

Your department:

Your speciality (or your degree):

1. (Clinical expert perspective): Which are the everyday practices that can increase and sustain the intrinsic capacity of the older population? Please, refer to everyday activities that each older person can do by him/herself such as monitoring health vital signs, taking appropriate medication, lifestyle activities, etc.
2. (Technology enhancement): Can you please indicate what kind of technology you would suggest supporting everyday practice to enhance Intrinsic capacity?
3. (Supporting IC during COVID-19): Which technologies were found as applicable, in your everyday practice, to support the functional ability and quality of life of older people, during the Covid-19 crisis?
4. (Gender issue): How do you consider gender differences when you plan an intervention to support intrinsic capacity and functional ability?
5. (Closing question): What are the positive and the negative aspects of the Intrinsic Capacity Framework? Could you suggest improvements and future areas of intervention to support older people?
